# Effects of front-of-package nutrition labelling systems on objective understanding and purchase intention in El Salvador: results from a multi-arm parallel-group randomised controlled trial

**DOI:** 10.1007/s00394-025-03626-9

**Published:** 2025-06-25

**Authors:** Fabio da Silva Gomes, Alexandra Gálvez, Danilo Pérez, Carolina Paz, Claudia Alfaro, José Gerardo Merino, Gastón Ares, Carlos Felipe Urquizar Rojas

**Affiliations:** 1 Pan-American Health Organization (PAHO), Washington, D.C USA; 2 Centro para la Defensa del Consumidor, San Salvador, El Salvador; 3 Universidad Centroamericana José Simeón Cañas, San Salvador, El Salvador; 4 Instituto de Nutrición de Centroamérica y Panamá (INCAP), Guatemala City, Guatemala; 5 Facultad de Química, Instituto Polo Tecnológico de Pando, Universidad de la República, Pando, Uruguay; 6 Department of Design, University of Joinville Region, Joinville, Brazil; 7 Laboratory of Information Design Systems (LabDSI), Federal University of Paraná, Curitiba, Brazil; 8 525 23rd St. NW, Washington, D.C 20037 USA

**Keywords:** Health policy, Nutrition & dietetics, Public health, Food labelling, Decision making, Food choice, Front-of-package labelling

## Abstract

**Purpose:**

Front-of-package labelling (FOPL) schemes have been developed to provide more useful information to consumers and facilitate more critical and healthier food choices. This single-blinded multi-arm parallel-group randomised controlled trial aimed at assessing the effect of different FOPL schemes on the objective understanding of the nutritional content and intention to purchase products, in El Salvador.

**Methods:**

Participants (*n* = 1204) were exposed to two-dimensional images of 15 mock-up products presented at random and balanced orders. Participants were exposed to mock-ups featuring no FOPL scheme (control group, *n* = 305) or one of the following schemes: black octagonal warning labels (OWL) (*n* = 302), traffic-light labelling (TFL) (*n* = 297), or guideline daily amounts (GDA) (*n* = 300).

**Results:**

The odds for choosing to purchase the least harmful of the options more often was the highest in the OWL group compared to the control group: two times higher in the OWL group (OR 2·21, 95% confidence interval 1·58 − 3·11), and 49% higher in the TFL (1·49, 95%CI 1·06 − 2·11), with no changes in the GDA (1·06, 95%CI 0·75 − 1·50). OWL also resulted in the highest odds for correctly identifying the least harmful option (OR 3·77, 95%CI 2·79 to 5·09), and for correctly identifying a product with higher amounts of sugars, sodium, total fat, and/or saturated fats (3·26, 95%CI 2·40 to 4·43).

**Conclusion:**

OWL outperformed GDA and TFL in its ability to improve objective understanding of nutritional information and purchase intention. Results support the adoption of OWL in El Salvador.

**Trial registration number:**

ISRCTN 12,389,597.

**Date of registration:**

17 July 2023 (retrospectively registered).

**Supplementary Information:**

The online version contains supplementary material available at 10.1007/s00394-025-03626-9.

## Background

El Salvador is the smallest and most population dense country in Central America, and one fourth of its population lives under poverty situation [1]. The expansion of unhealthy eating practices driven by ultra-processed products have been worsening the vulnerability of living conditions for the population by leveraging overweight, obesity, and diet-related noncommunicable diseases (NCD) [2–4].

Obesity and NCDs continue to rise sharply [3, 4], and sugar-sweetened beverages intake have been estimated to cost El Salvador in direct medical expenses a total of USD $69.35 million in 2020 [5]. In 2017, expenditures with type 2 diabetes and hypertension, which are closely linked to excessive intakes of sugars and sodium, accounted for 92.3% of health budget. Health costs due to obesity have been estimated at USD $800 million, which was equivalent to 3.2% of El Salvador’s Gross Domestic Product in 2017 [6].

For these reasons, public policies that can effectively (re)shape food environments to support and protect healthy diets are essential [7–9]. Food labels are usually designed putting greater emphasis and salience on commercial features and persuasive elements to drive consumers preference and consumption, often opposing a healthy food environment [10, 11]. In the absence of mandatory policies that require effective tools to help consumers make informed decisions, an important piece of enabling healthy food environments will be missing [12, 13]. Quantitative nutrient declaration, which is provided in many countries, require a deal of time and cognitive effort that is greater than what consumers can employ during food purchase decisions, hence this label element is seldom used [14–20].

Front-of-package labelling (FOPL) schemes have been developed worldwide attempting to provide more useful information to consumers and facilitate more critical and healthier food choices [21, 22]. Doing so with an equity lens to make them particularly helpful for consumers with lower education and lower functional nutrition knowledge [23].

Considering that almost half of deaths are caused by high blood fasting glucose, hypertension and overweight and obesity [24], one of the specific purposes set to be met with FOPL is to allow consumers to easily, quickly and correctly identify products that are excessive in nutrients associated with those risk factors, including sugars, total fat, saturated fats, trans fats, and sodium, and to discourage their consumption [25, 26]. For this reason, research exploring which scheme can fit best this purpose has been critical to inform policy development.

El Salvador, like other Central American countries, has put forward a proposal of a FOPL system, which has been tabled in the parliament [27]. The production of local evidence could help informing the development and adoption of regulatory policies at national and subregional levels and worldwide.

This study compares the octagonal warning labels (OWL) included in the proposal put forward by the Council of the Ministers of Health of Central America and Dominican Republic (COMISCA) for adoption by the Central American Integration System [28], which coincides with the one proposed by legislators in the Salvadorean domestic parliament, and the guideline daily amounts (GDA) and the traffic-light labelling (TFL), which have been proposed by food industry sectors as alternatives.

The objectives of the study were aligned with the regulatory objectives sought to be met by COMISCA with FOPL in Central America and Dominican Republic [28]. The trial aimed at assessing the effects of these FOPL schemes on the objective understanding of the nutritional content (correctly identifying the least harmful option, correctly identifying sugars, sodium and/or saturated fats found to be in excess) and on the choice to purchase the least harmful option (purchase intention) of a series of products.

## Methods

### Design

A single-blinded multi-arm parallel-group randomised controlled trial was conducted among adults in El Salvador. Participants were randomly allocated at equal rate (1/4) to the four study groups (three experimental and the control group). Participants in each group were exposed to either one of the experimental conditions or assigned to the control group.

## Participants

**Fig. 1:**
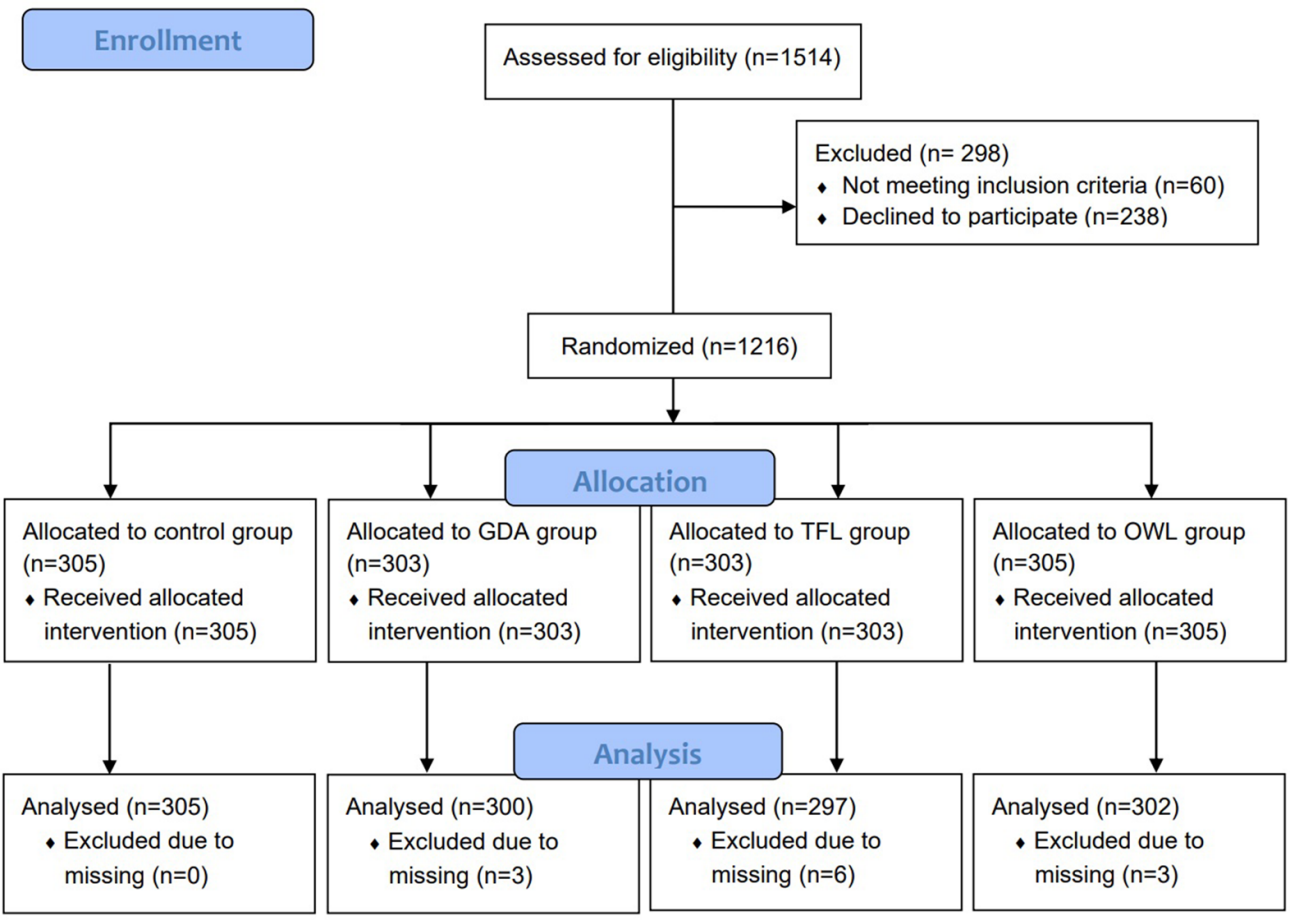
CONSORT flow diagram

Adults residing in El Salvador (*n* = 1204) (Fig. 1) with 18 years old or older were included in the study, except for those visually impaired, or unable to read or to give informed consent. Residents from all 14 provinces of the country were interviewed. Households located in the surroundings of popular supermarkets were selected using a two-stage quota sampling design. Interviewers followed a walking path of travel starting from the supermarket to select the households. Households were screened for the quota conditions (first stage), and then from each household, one adult responsible for grocery shopping was interviewed (second stage). Household screening and interviews were conducted until the number of successful interviews needed was reached. Participants were invited by interviewers to participate in a survey about food packages, they were informed that the study aimed to find out how people in El Salvador perceive food packages, and that they would be presented with a series of pictures of food packages and asked to answer simple questions that would take about 15 min to respond in total.

### Interventions

**Fig. 2:**
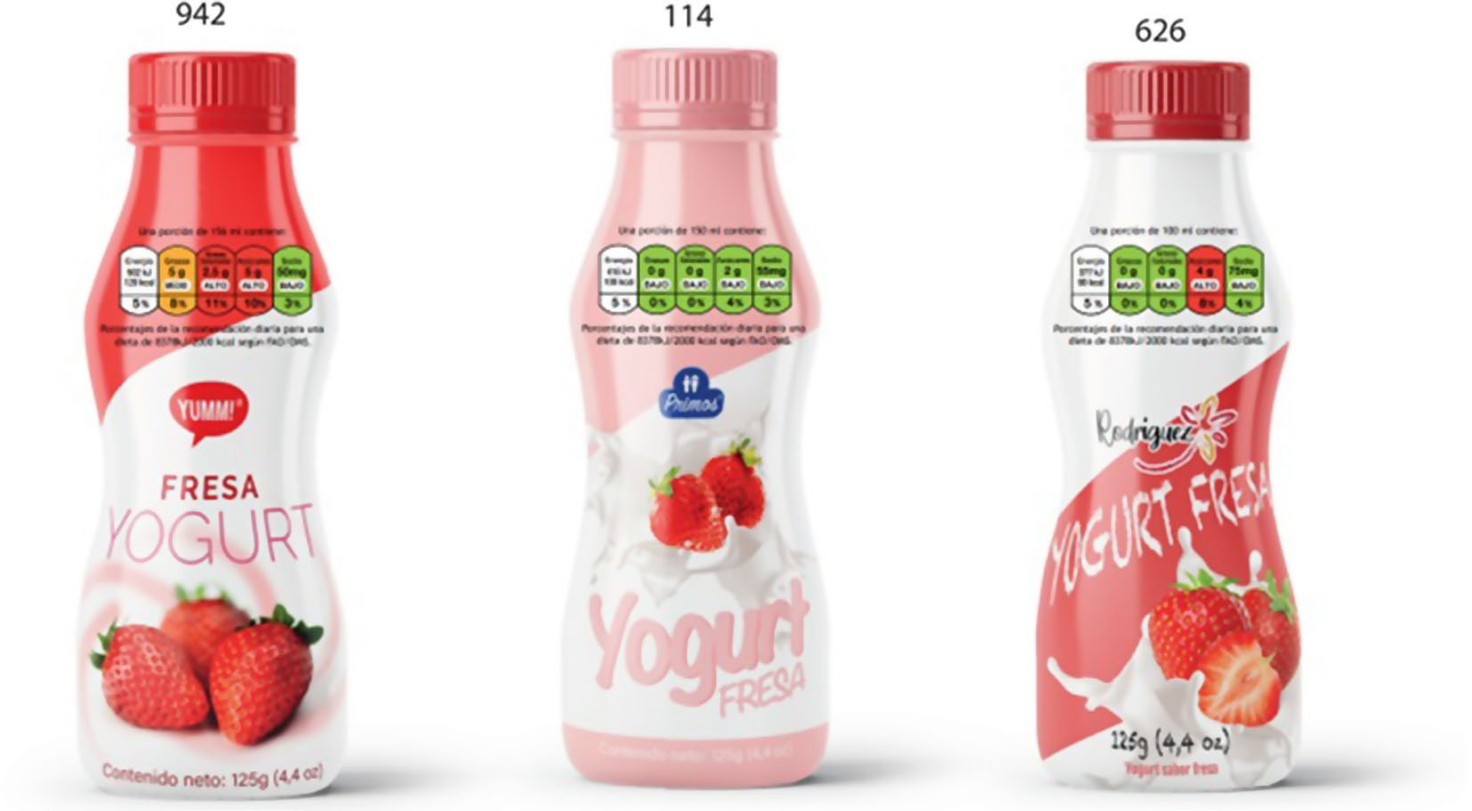
Example of a set of images of one category of products shown to participants with one of the FOPL schemes tested (TFL)

Participants were shown two-dimensional (2D) images of 15 different mock-up products presented at random and balanced orders between and within categories of products. The images were printed in A3 size booklets. Figure 2 illustrates one of the pages of a booklet of images shown to participants of one of the experimental groups.

The mock-ups resembled characteristics of real commercial products available in the Salvadorean market in terms of package and graphic design, and nutritional composition. Five sets of mock-ups were designed. Each set included three products from each of five product categories of ultra-processed products commonly consumed (3 × 5 = 15 mock-up products). The product categories were breakfast cereal extrudates, chocolate flavoured milks, filled cookies, white breads, and yoghurts.

The same 15 mock-up products were used in each group; the only difference across groups was the FOPL scheme they were featuring. Participants were randomly allocated to one of four experimental groups: OWL, TFL, GDA, or no FOP label (control group). Mock-ups shown to participants featured solely the scheme they were allocated to.

**Fig. 3:**
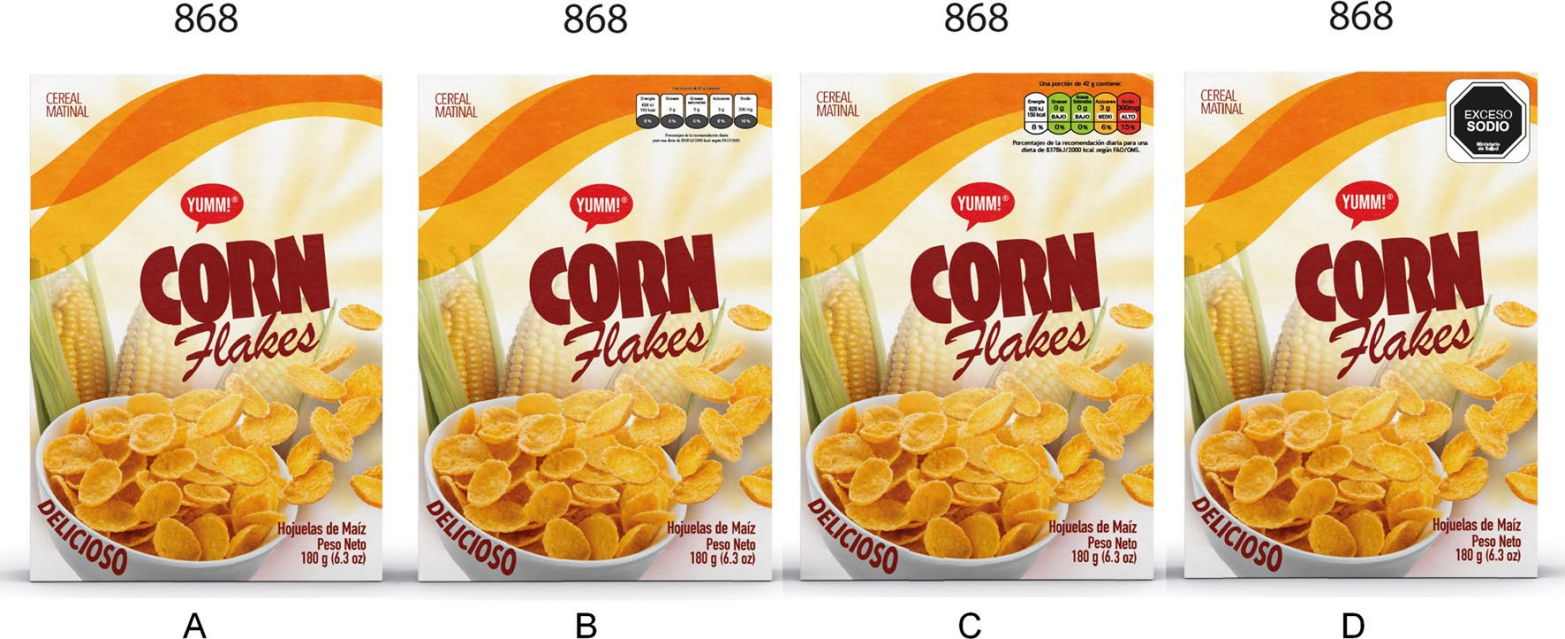
Example of a product with the different front-of-package labelling (FOPL) schemes applied. **A**: no FOPL (control condition); **B**: guideline daily amounts (GDA); **C**: traffic-light labelling scheme (TFL); **D**: octagonal warning label (OWL)

The application of TFL followed the specifications developed by the UK Department of Health, the Food Standards Agency, and devolved administrations in Scotland, Northern Ireland and Wales in collaboration with the British Retail Consortium [29]. For the GDA the specifications proposed by industry for adoption by the in Central American Integration System were used [30]. The specifications used for the application of black octagonal warning labels followed the standard proposed by COMISCA [28]. For consistency, thresholds used to define the ‘high/excess’ content of sugars, fats, saturated fats, or sodium, were the same for all FOPL systems, when such category applied (i.e. OWL and TFL), and the Pan American Health Organization criteria included in the COMISCA proposed standard was the one used [31]. See the nutritional composition of products in the supplementary material (Table S1). All sets of mock-up products were identical except for the FOPL icons featured. Figure 3 illustrates one of the mock-up products with the FOPL schemes applied.

## Randomisation

Participants were selected using quota sampling to meet a composition of age, sex, and educational level within each group that resembles the one found for the population of El Salvador. A similar number of participants were randomly allocated to one of the three intervention groups or the control group: OWL (*n* = 302); TFL (*n* = 297); guideline daily amounts GDA (*n* = 300); and the control group which was not exposed to any FOPL scheme (*n* = 305). The randomisation of the experimental conditions and groups was completed adopting a Williams design [32] to ensure the order of categories of products and the order of products within categories was random and balanced for all groups. This randomisation produced five random and balanced sequences of categories of products and products within categories used for each of the four groups, resulting in 20 possible combinations of groups and sequences (5 × 4), equally balanced and order within each group.

For the allocation of participants into each of the groups and each of the five sequences within the group, a simple randomisation technique was adopted using a Pareto randomisation procedure. Random numbers falling between 0 and 1 were generated for each of the 20 combinations of groups and sequences, then these combinations were sorted by the random numbers and used to sequentially randomise participants’ allocation to one of these 20 combinations of groups (experimental or control) and random sequences of categories of products and of products within categories.

The study was single-blinded, since participants were not aware they had been assigned to an intervention or control group. Although field researchers had no prior knowledge of which intervention would be assigned to a participant, once, and only once, the exposure of the participant to an intervention or the control was initiated, researchers could tell which study group participants were assigned to.

## Experimental procedure

Participants were interviewed by trained field researchers after providing their signed informed consent. The recruitment and interviews took place in their homes, on weekdays and weekends, from 01-02-2022 to 14-02-2022. The order of the questions asked aimed at reducing potential response bias. For the first task, participants were shown the three products from each of the five product categories and asked to indicate which product they would buy in each category. The five product categories were shown one by one. Both the categories and the products within the categories were presented in random and balanced order.

For the second task, participants were shown the same products now in different order of categories, and different order within categories. For each set of three products within a category they were asked to indicate which of those was the least harmful for health.

For the last task, participants were shown one product of each category, in a random and balanced order, and asked if the product had an amount of sugars, sodium, total fat, saturated fat, trans fat, or none of these nutrients, that was higher than the recommended for a healthy diet.

Finally, participants answered questions about their sociodemographic and health statuses.

### Outcomes

The primary outcomes of the study included the contribution of the different FOPL schemes to improving the decision of participants to buy the least harmful option more often, the correct identification of the least harmful option more often, and the correct identification of sugars, sodium, total fat, trans fats, and/or saturated fats found to be in excess in the products more often.

For the first task participants had five opportunities to indicate which product, out of a set of three, they would buy, or whether they would not buy any of the products, which was used to estimate the frequency with which participants intended to buy the least harmful option. The second task also provided five opportunities for participants to identify the least harmful product, out of a set of three, and this data were used to estimate the frequency with which participants made a correct identification. The third task allowed participants to verify five products, one at a time, and to indicate whether they contained amounts of sugars, sodium, total fat, trans fats, and/or saturated fats found to be higher than the recommended for a healthy diet. With these data the number of correct answers and the proportion of participants with zero, one, two, three, four or five correct answers for the total of five products was estimated.

These estimated frequencies were compared across experimental and control groups to assess how FOPL schemes performed according to the primary outcomes described above.

## Sample size

The sample size was estimated based on calculations to detect a difference between two proportions. The most conservative criterion was used, assuming that the proportion of participants who correctly identify products with nutrient above nutritional recommendations for the control condition would be 50%. The number of participants needed to detect an absolute increase of 12% (which is smaller than what has been previously reported [33, 34]) with a confidence level of 95% and a power level of 80% was estimated in 265 participants in each of the experimental groups (comparisons and control) (*n* = 265 × 4 = 1060). A total of 1216 adults completed the interview, twelve respondents were removed due to missing data on age, education, or sex (1%). The final resulting sample used was of 1204 participants.

## Statistical analyses

Descriptive statistics on the sample included proportions (for categorical variables), means (for numeric variables) and their respective 95% confidence intervals (95%CI). To examine the contribution of FOPL schemes to improving the frequency with which consumers would intend to purchase the least harmful options of products, ordered logistic regression models were used to estimate the odds ratio (OR) of choosing to purchase the least harmful products more often. The number of times participants have indicated they would purchase the least harmful option was the ordinal dependent variable, and FOPL schemes the independent one. To analyse the contribution of FOPL schemes to the correct identification of the least harmful options of products, a similar model was adjusted. The number of times participants have correctly identified the least harmful option was the ordinal dependent variable, in this case. Following a similar procedure, the number of times participants correctly identified when products contained excessive amounts of sugars, total fat, saturated fats, trans fats and/or sodium was analysed as the ordinal dependent variable to verify the contribution of FOPL schemes in helping consumers to correctly identify those more often. The odds ratios (OR) respective 95%CI were also calculated, and Wald test was used to verify the significance of the contribution of FOPL schemes against the control condition and between schemes for the three outcomes listed above.

Models were adjusted for age, sex, and education level. A similar set of models were also adjusted using logistic regression with logit link function for each single category of products as subset analyses.

All tests were two-sided, and we considered *p* < 0.05 to be statistically significant. The analyses were conducted in R language and environment for statistical computing version 4.0.1 [35].

## Results

Figure 1 shows the numbers of participants who were randomly assigned, received the intended intervention, and were analysed for the primary outcome. As shown in Table 1, most of the respondents were under 50 years of age, women, and reached the secondary level of education or lower. In addition, the most common reported noncommunicable disease condition and risk factor was hypertension, followed by diabetes, high cholesterol, overweight and obesity, and heart disease.

**Table 1:** Sociodemographic characteristics and reported noncommunicable diseases conditions and related risk factors of the sample

	Total (*n* = 1204)	Control (*n* = 305)	GDA (*n* = 300)	TFL (*n* = 297)	OWL (*n* = 302)
**Age brackets**					
18–30	361 (30.0%)	86 (28.2%)	99 (33.0%)	80 (26.9%)	96 (31.8%)
31–50	488 (40.5%)	135 (44.3%)	112 (37.3%)	118 (39.7%)	123 (40.7%)
51–70	302 (25.1%)	74 (24.3%)	68 (22.7%)	87 (29.3%)	73 (24.2%)
71+	53 (4.4%)	10 (3.3%)	21 (7.0%)	12 (4.1%)	10 (3.3%)
**Women**	829 (68.9%)	208 (68.2%)	205 (68.3%)	209 (70.4%)	207 (68.5%)
**Men**	375 (31.1%)	97 (31.8%)	95 (31.7%)	88 (29.6%)	95 (31.5%)
**Educational levels**					
Primary school or lower	231 (19.2%)	53 (17.4%)	72 (24.0%)	57 (19.2%)	49 (16.2%)
Secondary	783 (65.0%)	216 (70.8%)	176 (58.7%)	187 (62.9%)	204 (67.6%)
Tertiary	190 (15.8%)	36 (11.8%)	52 (17.3%)	53 (17.9%)	49 (16.2%)
**Participants who have been informed by a health professional that they have**					
Diabetes or raised blood sugar	347 (28.8%)	77 (25.2%)	97 (32.3%)	84 (28.3%)	89 (29.5%)
Hypertension or high blood pressure	391 (32.5%)	87 (28.5%)	108 (36.0%)	92 (31.0%)	104 (34.4%)
Heart disease	154 (12.8%)	41 (13.4%)	39 (13.0%)	33 (11.1%)	41 (13.6%)
High cholesterol	259 (21.5%)	69 (22.6%)	64 (21.3%)	61 (20.5%)	65 (21.5%)
Overweight or obesity	231 (19.2%)	58 (19.0%)	62 (20.7%)	51 (17.2%)	60 (19.9%)

GDA: guideline daily amounts; TFL: traffic-light system; OWL: octagonal warning labels

When compared to the control, the chances of participants choosing to purchase the least harmful option more often doubled when they were exposed to the OWL (OR 2·21, 95%CI 1·58 to 3·11). The TFL performed significantly better than the control condition, but worse than the OWL, whereas the GDA (1·06, 95%CI 0·75 to 1·50) was inefficacious in improving such odds compared to the control condition (Table 2).

**Table 2:** Effect of different FOPL schemes on the objective Understanding of the nutritional content, harmfulness perception, and intention to purchase products, in El Salvador, compared to the control condition.^‡^ values are odds ratios (95% confidence intervals)

		Front-of-package labelling experimental groups		
Outcomes	Products	GDA (*n* = 300)	TFL (*n* = 297)	OWL (*n* = 302)
Intention to purchase the least harmful option	All categories of products	1.06 (0.75;1.50) ^a^	1.49 (1.06; 2.11)* ^b^	2.21 (1.58; 3.11)* ^c^
	Breakfast cereals	1.14 (0.75; 1.73) ^a^	1.41 (0.94; 2.13) ^a^	2.27 (1.54; 3.35)* ^b^
	Yogurts	0.64 (0.39; 1.02) ^a^	1.39 (0.91; 2.12) ^b^	3.53 (2.40; 5.26)* ^c^
	Flavoured milks	0.85 (0.59; 1.22) ^a^	0.96 (0.67; 1.38) ^a, b^	1.30 (0.92; 1.84) ^b^
	Filled cookies	1.20 (0.78; 1.83) ^a, b^	0.93 (0.60; 1.44) ^a^	1.61 (1.08; 2.41)* ^b^
	White breads	1.06 (0.71; 1.59)	1.36 (0.92; 2.02)	1.47 (1.00; 2.16)*
Correct identification of the least harmful option	All categories of products	0.92 (0.69; 1.23) ^a^	2.39 (1.78; 3.20)* ^b^	3.77 (2.79; 5.09)* ^c^
	Breakfast cereals	0.77 (0.54; 1.09) ^a^	1.44 (1.03; 2.02)* ^b^	2.32 (1.67; 3.24)* ^c^
	Yogurts	0.98 (0.71; 1.36) ^a^	2.06 (1.48; 2.88)* ^b^	2.72 (1.94; 3.84) * ^b^
	Flavoured milks	0.86 (0.62; 1.20) ^a^	1.68 (1.20; 2.36)* ^b^	1.81 (1.29; 2.55)* ^b^
	Filled cookies	1.18 (0.84; 1.65) ^a^	1.48 (1.07; 2.07)* ^a, b^	1.94 (1.40; 2.70)* ^b^
	White breads	1.09 (0.76; 1.58) ^a^	2.50 (1.76; 3.55)* ^b^	3.33 (2.36; 4.73)* ^b^
Correct understanding about the nutritional content of products	All categories of products	0.96 (0.71; 1.31) ^a^	1.63 (1.20; 2.21)* ^b^	3.26 (2.40; 4.43)* ^c^
	Breakfast cereals	1.50 (1.00; 2.26)* ^a^	1.60 (1.07; 2.41)* ^a^	2.85 (1.95; 4.20)* ^b^
	Yogurts	0.57 (0.36; 0.90)* ^a^	1.49 (1.01; 2.21)* ^b^	1.72 (1.18; 2.53)* ^b^
	Flavoured milks	1.03 (0.59; 1.81) ^a^	1.14 (0.66; 1.97) ^a^	2.31 (1.43; 3.80)* ^b^
	Filled cookies	0.95 (0.47; 1.92) ^a^	0.56 (0.24; 1.23) ^a^	2.08 (1.15; 3.91)* ^b^
	White breads	0.58 (0.36; 0.92)* ^a^	2.08 (1.41; 3.08)* ^b^	4.21 (2.91; 6.17)* ^c^

^‡^Estimates for sets of products were obtained using ordered logistic regression models and estimates for single categories of products were obtained using logistic regression models with link function binomial logit. All estimates were adjusted for adjusted for age, sex, and education level

*Significantly different from control condition. Also highlighted in bold (*p* ≤ 0.05)

^a, b,c^ Different superscript letters within a row in the comparison between columns indicate significant differences between the effects of FOPL schemes (*p* ≤ 0.05)

When data were analysed for each product category separately, the effects exerted by the OWL on the intention to purchase the least harmful option were similar for almost all separate product categories. However, the TFL and GDA were inefficacious in improving such outcome for every separate food category (i.e. breakfast cereals, yogurts, flavoured milks, filled cookies, and white breads) (Table 2).

The odds of participants correctly identifying the least harmful option more often was the highest and almost quadrupled compared to the control when they were exposed to the OWL (OR 3·77, 95%CI 2·79 to 5·09). Again, the TFL performed significantly worse than the OWL, and the GDA (0·92, 95%CI 0·69 to 1·23) was inefficacious in improving such odds, compared to the control (Table 2).

When analysing the results separately by product category, the OWL again performed best in improving the capacity of participants to identify the least harmful option for all product categories. The GDA was unable to help participants completing this task correctly for all categories (Table 2).

**Fig. 4:**
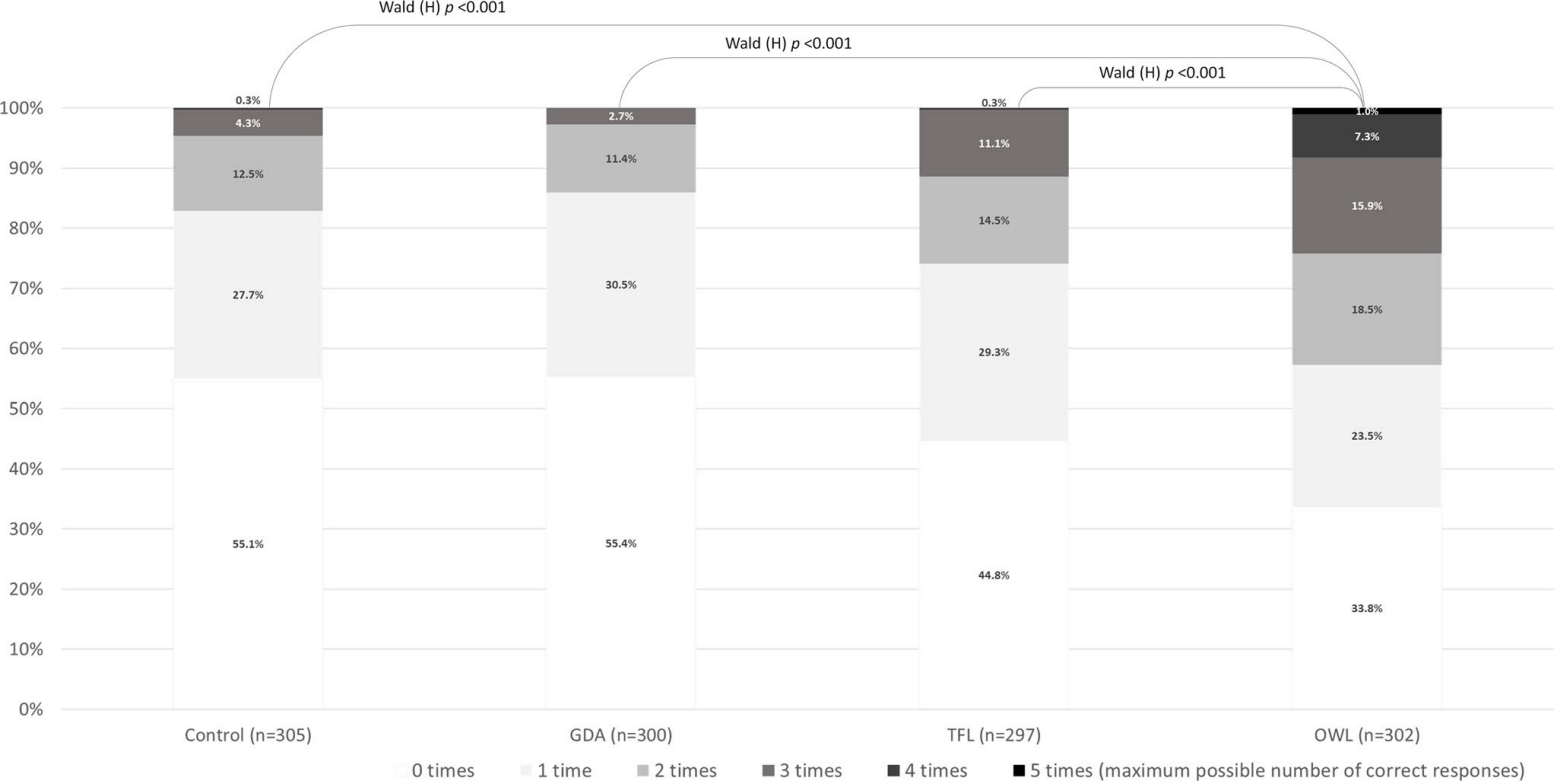
Proportional distribution of the number of times participants correctly identified the presence of amounts of critical nutrients higher than recommended for a healthy diet in products, by experimental groups. Wald (H): Wald statistics for homogeneity indicating proportional distributions differ significantly. GDA: guideline daily amounts; TFL: traffic-light labelling scheme; OWL: octagonal warning label

The chances of participants correctly identifying when a product contained amounts of critical nutrients (sodium, sugars, total fat, saturated fats) higher than recommended for a healthy diet tripled when they were exposed to the OWL (3·26, 95%CI 2·40 to 4·43). The TFL performed significantly worse than the OWL, whereas the GDA (0·96, 95%CI 0·71 to 1·31) was inefficacious (Table 2). The Wald statistics for homogeneity also confirms the superiority of OWL in improving the capacity of participants to correctly identify products with amounts of critical nutrients higher than recommended for a healthy diet (Fig. 4). When analysing these results separately by product category, the OWL performed best for all product categories. The GDA was only able to help participants completing this task correctly when applied to breakfast cereals. In addition, it worsened the capacity of participants correctly completing this task for yogurts and white breads (Table 2).

## Discussion

OWL performed better than the GDA and TFL in helping consumers correctly identifying the least harmful option, the presence of higher amounts of critical nutrients, and choosing to purchase the least harmful option in El Salvador, regardless of the populations’ age, sex, and education level.

Previous studies with similar findings, have reported that warning labels are easier to understand and perform better in improving purchase intentions than GDA and TFL [30, 33, 34, 36–43]. In this study GDA was inefficacious in helping consumers make healthier choices.

GDA functions as a miniature of quantitative nutrient declaration tables, for this reason it presents similar limitations in helping improving consumers decision. It does not provide any interpretation to the numeric information it presents, and as a result it also does not address the limitations and disparities associated with the understanding of numeric nutrient information [9]. Deliza et al. (2019) reported that consumers exposed to the GDA required significantly more time to detect the presence of nutrients in excess in products given this scheme’s lack of interpretational aids [37]. Arrúa et al. (2017) found similar results, and in addition reported that GDA performed worse than OWL in improving consumers’ ability to identify unhealthful products and correct misperceptions about the healthfulness of products excessive in sodium, sugars, saturated fats [33].

In this trial, TFL performed better than GDA, which has also been reported by some previous studies [34, 37]. That is likely because TFL adds interpretative aids to GDA, including textual information and colour coding borrowed from traffic lights. However, when compared to OWL, TFL performed significantly worse for all outcomes of this trial. The colourful icon of TFL makes it less salient than OWL, when applied to the also colourful labels of processed and ultra-processed products. In addition, colours exert psycho- and neurophysiological effects on consumers when associated with edible and drinkable products, which differ from those intended to communicate traffic orientation, and may elicit undesirable effects [36, 44–51]. Previous studies have reported that the green colour may drive consumers to perceive products as healthier [36, 44, 46, 48], and that the red colour triggers greater appetite for sweet ultra-processed products [51]. In addition, the TFL may signal conflicting valence in some products, as they simultaneously feature different colour codes and concentration levels for different nutrients: high (red colour), medium (amber colour), and low (green colour). These features may help explaining the findings on TFL inefficacy or lower efficacy for improving food purchase decisions [33, 34, 38, 40–43, 52].

No previous study has found TFL performed better than OWL, but in contrast with our findings, two studies conducted in a virtual setting were not able to detect significant differences between OWL and TFL’s effects on purchase intention [53, 54]. Nevertheless, one of them reported warnings performed better than TFL in improving the understanding of nutritional content [54].

Consistent with the findings of this trial, impact studies conducted after the implementation of OWL in Chile and Uruguay have reported an increase in objective understanding and changes in purchase decisions [55–58]. In particular, an interrupted time series found warning labels contributed to reducing the purchase of products high in calories, sodium, saturated fats, and sugars in Chile [58].

The present study has strengths and limitations. Its multi-arm parallel-group randomised controlled design avoids differential carry-over effects which are an important limitation of within-subject studies. The use of 2D mock-up products standardised to match real products’ and resemble real-life application of FOPL strengthened the trial external validity. Conversely, the study was conducted in a household and not a supermarket setting which could have weakened this validity. The absence of price information and the use of fictitious brands have strengthened the clear attribution of the effects to the FOPL schemes, reciprocally this has limited the analysis of the potential contribution of these factors to explain the variations on the outcomes. Further experimental research on the efficacy of the evaluated FOPL schemes in a real supermarket setting with a complete assortment of products would be useful, particularly for the schemes that have not been evaluated in real-life situations (i.e. GDA and TFL).

Finally, it should be acknowledged that FOPL is not expected to function as a silver bullet for all public health nutrition issues, but rather one policy tool to be considered within a comprehensive package of strategies needed to transform food systems and promote healthier and more sustainable diets [8, 22].

## Conclusion

The findings of this study indicate that the octagonal warning labels included in the COMISCA proposal [28] to be adopted by Member States of the Central American Integration System and in bills to be adopted by the Salvadorean parliament is the most effective option in meeting the regulatory objective of helping the population to correctly identify the presence of excessive amounts of critical nutrients, and to correctly identify and to choose to purchase the least harmful products, more often.

## Electronic supplementary material

Below is the link to the electronic supplementary material.

Supplementary Material 1
