# Supplementary material for: Effects of front-of-package nutrition labelling systems on objective understanding and purchase intention in El Salvador: results from a multi-arm parallel-group randomised controlled trial
Source: Eur J Nutr. 2025 Jun 25;64(5):228. doi: 10.1007/s00394-025-03626-9 (PMC12198295; doi:10.1007/s00394-025-03626-9)
Supplement: Supplementary file 1 — Supplementary Material 1 [file 394_2025_3626_MOESM1_ESM.docx]

**Supplementary material**

**Table S1 – Nutritional composition of mock-up products.**

| **Products** | **Serving size** | **Energy (kJ/kcal)** | **Total fat (g)** | **Saturated fat (g)** | **Sugars (g)** | **Sodium (mg)** |
| --- | --- | --- | --- | --- | --- | --- |
| Filled cookies 497 | 40g | 817/195 | 9 | 5.5 | 11.8 | 109 |
| Filled cookies 516 | 42g | 817/195 | 8 | 4.3 | 13.1 | 94 |
| Filled cookies 695 | 15g | 712/170 | 5.6 | 2.3 | 14 | 160 |
| Yogurt 114 | 150g | 418/100 | 0 | 0 | 2 | 55 |
| Yogurt 626 | 100g | 377/90 | 0 | 0 | 4 | 75 |
| Yogurt 942 | 156g | 502/120 | 2.5 | 1.5 | 5 | 50 |
| Cereal 351 | 28g | 460/110 | 0 | 0 | 9 | 170 |
| Cereal 868 | 42g | 628/150 | 0 | 0 | 4 | 300 |
| Cereal 964 | 30g | 473/196 | 0.6 | 0 | 3 | 190 |
| Flavored milk 215 | 237ml | 628/150 | 2.5 | 1.5 | 11 | 130 |
| Flavored milk 301 | 200ml | 699/167 | 5.1 | 2.4 | 14 | 160 |
| Flavored milk 566 | 190ml | 711/170 | 5.5 | 3 | 13 | 130 |
| White bread 143 | 43g | 493/118 | 1.5 | 1 | 1 | 220 |
| White bread 405 | 23.33g | 268/64 | 1.2 | 0.6 | 0.7 | 100 |
| White bread 830 | 18.46g | 209/50 | 0.7 | 0.3 | 0.8 | 49 |
